# The Relationship between Selenium and T3 in Selenium Supplemented and Nonsupplemented Ewes and Their Lambs

**DOI:** 10.1155/2014/105236

**Published:** 2014-02-10

**Authors:** Abd Elghany Hefnawy, Seham Youssef, P. Villalobos Aguilera, C. Valverde Rodríguez, J. L. Tórtora Pérez

**Affiliations:** ^1^Faculty of Veterinary Medicine, Benha University, Moshtohor 13736, Egypt; ^2^Instituto de Neurobiología, Universidad Nacional Autónoma de México, Mexico; ^3^Facultad de Estudios Superiores Cuautitlán, Universidad Nacional Autónoma de México, Km. 2.5 Carretera Cuautitlán-Teoloyucan, 54714 Cuautitlán Izcalli, MEX, Mexico

## Abstract

Twenty pregnant ewes were selected and classified into two groups. The first group received subcutaneous selenium supplementation (0.1 mg of sodium selenite/kg BW) at the 8th and 5th weeks before birth and 1st week after birth while the other was control group without selenium injection. Maternal plasma and serum samples were collected weekly from the 8th week before birth until the 8th week after birth and milk samples were taken from ewes weekly, while plasma and serum samples were collected at 48 hours, 1st, 2nd, 3rd, 5th, and 8th weeks after birth from the newborn lambs. Results demonstrated significant positive relationship between maternal plasma selenium and serum T3 in supplemented and control ewes (*r* = 0.69 to 0.72, *P* < 0.05). There was significant (*P* < 0.001) increase in T3 in supplemented ewes and their lambs until the 8th week after birth. There was positive relationship between milk, selenium concentration, and serum T3 in the newborn lambs of the supplemented group (*r* = 0.84, *P* < 0.01), while the relationship was negative in the control one (*r* = −0.89, *P* < 0.01). Muscular and thyroid pathological changes were independent of selenium supplementation. Selenium supplementation was important for maintaining T3 in ewes and newborn lambs until the 8th week after birth.

## 1. Introduction

Recognized as an essential trace element in 1957 [1], selenium (Se) is a key component of the so-called selenoproteins and plays a critical role in various aspects of human and animal health [2, 3]. In the thyroid gland, Se is associated with the activity of redox-protective peroxidases which prevent thyroid cell from oxidative damage during the process of hormonogenesis [4, 5]. Furthermore, Se is also necessary in the synthesis of iodothyronine deiodinases (D), the family of selenoenzymes which are critical for the control of thyroid hormone (TH) action at the cellular level. Deiodinases type 1 and 2 (D1 and D2) catalyze the activation of tetraiodothyronine (T_4_) to triiodothyronine (T_3_) and D1 and D3 inactivate T_4_ to reverse T_3_ or T_3_ to T_2_ [2, 6, 7]. Se deficiency induces a significant reduction in T_3_ with the corresponding increase in T_4_ and a reduction in the activity of hepatic D1 [2, 8–13]. On the other hand and as recently reviewed [3], several studies have shown that supplemental sodium selenite and sodium selenate by oral or parenteral administration forestall the clinical signs of Se deficiency and animal losses in ruminant and nonruminant species [14, 15]. Transfer of nutrients and Se from the dam to the offspring occurs via two pathways, placental transfer and colostrum-milk ingestion. The amount of nutrients transferred to offspring depends on the maternal nutrient status and the efficiency of the transplacental and mammary transport mechanism. Se is efficiently transferred via the placenta to the fetus, even in situations of low maternal concentration of the element [16, 17]. The objective of this study is to assess the effect of pre- and postpartum Se supplementation on thyroid and Se homeostasis in ewes and their newborn lambs.

## 2. Materials and Methods

**Figure 1:**
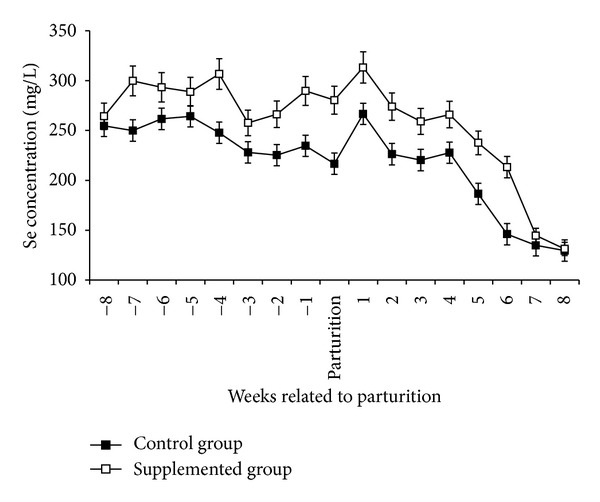
Plasma Se concentrations (mean ± SEM) of Se supplemented and control ewes.

Twenty primiparous pregnant Pelibuey ewes were selected after ultrasound examination approximately at 90 days of pregnancy. The ewes were 1.5–2 years of age with an average body weight of 41.09 ± 0.8 kg. These ewes were divided into two groups, the 1st group (*n* = 10) were supplemented with subcutaneous injection (SC) of sodium selenite, 0.1 mg/kg BW, at the 8th and 5th weeks prepartum and at the 1st week postpartum, while the control group (*n* = 10) did not receive Se. Deficiency of blood Se level in the experimental ewes was 250 ppm (Figure 1). Ewes had a high corporal condition (average body weight was 41.09 ± 0.8 kg). Both groups feed a diet elaborated with alfalfa, corn, and soybean with a mineral salt containing 0.5 iodine mg/kg DM and without Se (Table 1) and water ad libitum. The Se concentration in the diet was 0.22 and 0.23 ppm during pregnancy and lactation, respectively.

Blood samples from gestated ewes were collected weekly from the 8th week prepartum until the 8th week postpartum. Postpartum samples from newborns were obtained at 48 h and at the 1st, 2nd, 3rd, 5th, and 8th weeks and milk samples were collected weekly. In both ewes and newborns, blood was collected in the morning (8:00 am) by jugular vein puncture using vacutainer tubes and divided into two portions. The first portion was taken on EDTA as anticoagulant for obtaining of plasma while the second portion was left to clot at room temperature for about 20 minutes and then centrifuged at 3000 r.p.m. for 15 minutes; the supernatant serum samples were drown and kept frozen (−80°C) until analyzed. Milk (2 mL) was collected in the same schedule of the blood samples. As previously described, Se was measured in the plasma, milk, and diet by atomic absorption spectrophotometer [17], while serum T_3_ was measured by radioimmunoassay [18]. At the end of the experiment, ewes and lambs were sent to slaughterhouse and part of the thyroid gland, the diaphragmatic muscle, and the myocardium were collected in 10% buffered formalin solution and processed for histopathological evaluation to obtain paraffin cuts as routine.

## 3. Statistical Analysis

The obtained results from the experiments were expressed as mean ± SEM and were analyzed by analysis of variance (ANOVA) for repeated measures with means tested for significance by Duncan's multiple range tests. The correlation between the obtained results was tested with a Pearson correlation test. Differences were declared significant at *P* < 0.05.

## 4. Results

**Figure 2:**
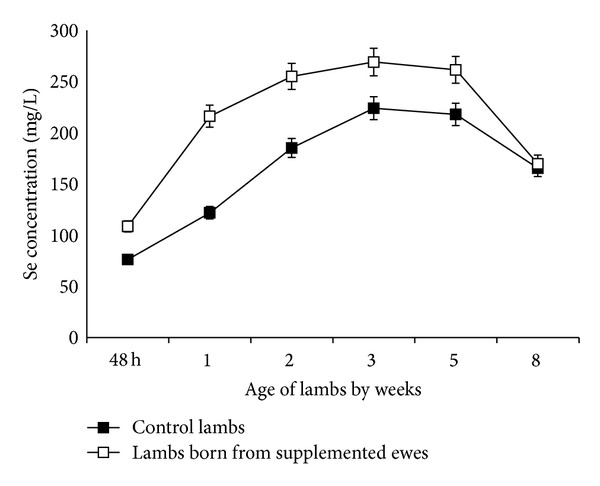
Plasma Se concentrations (mean ± SEM) of newborn lambs from Se supplemented and control ewes.

**Figure 3:**
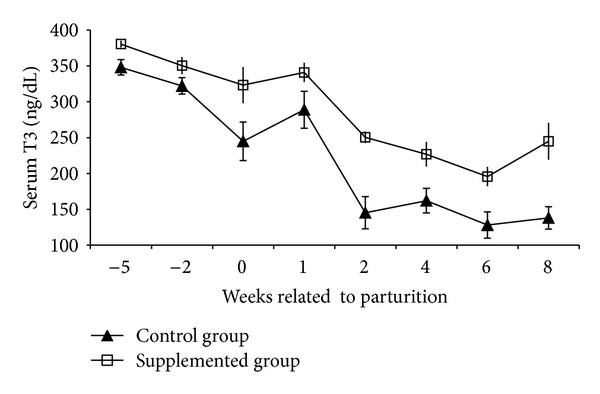
Serum T3 concentrations (mean ± SEM) of Se supplemented and control ewes.

**Figure 4:**
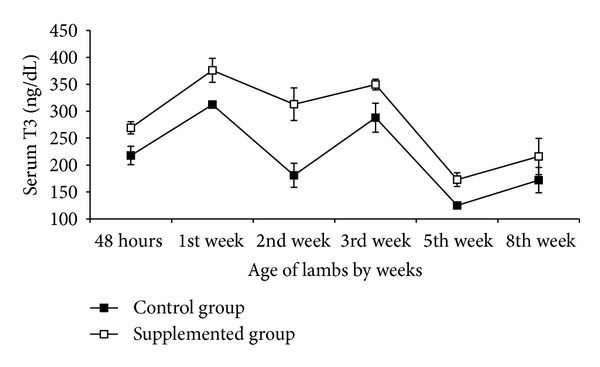
Serum T3 concentration (mean ± SEM) of newborn lambs from Se supplemented and control ewes.

One ewe from the control group aborted and was eliminated. There was significant (*P* < 0.05) increase in the plasma Se concentrations in Se supplemented ewes (Figure 1) and their lambs (*P* < 0.01) (Figure 2) than that of the nonsupplemented group. Compared to control animals and during the entire study, circulating levels of T_3_ were higher (*P* < 0.001) in Se supplemented ewes. Moreover, these differences in serum hormone concentrations became more evident during lactation where T_3_ mean values for supplemented and nonsupplemented ewes were 200 ± 60 ng/dL and 150 ± 35 ng/dL, respectively (Figure 3). Similarly, serum T_3_ in lambs born from Se supplemented ewes was higher (*P* < 0.01) than in those born from control ewes (Figure 4). This difference between both groups of newborns was maintained up to the 8th week of the age. There was a positive correlation between maternal plasma Se concentration and serum T_3_ in both supplemented and nonsupplemented ewes (*r* = 0.69 to 0.72, *P* < 0.05). In contrast, serum T_3_ and milk Se concentrations were positively correlated in the supplemented ewes (*r* = 0.60, *P* < 0.05), while this correlation was negative in the control group (*r* = −0.80, *P* < 0.05). A similar pattern between serum T_3_ and milk Se concentration was observed in newborns. The correlation was positive in the lambs born from Se supplemented ewes (*P* < 0.01, *r* = 0.84) and negative in the lambs born from the control group (*P* < 0.01, *r* = −0.89).

**Figure 5:**
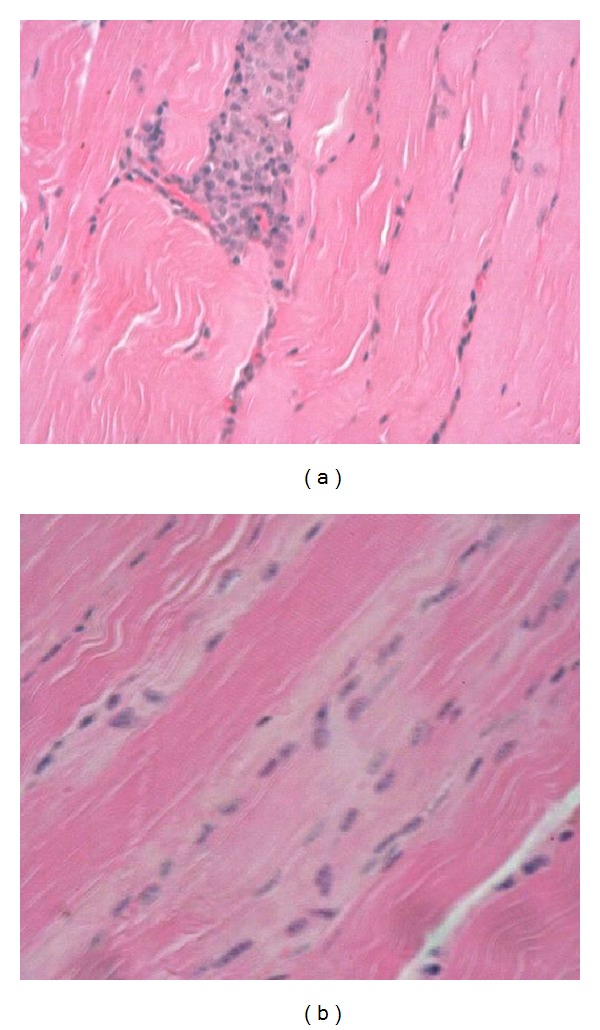
Muscular dystrophy. (a) Mononuclear infiltration replaced a damage muscular fiber, H&E, 160x. (b) Muscular nucleus proliferation in damage pale fibers in ewes. H&E, 80x.

**Figure 6:**
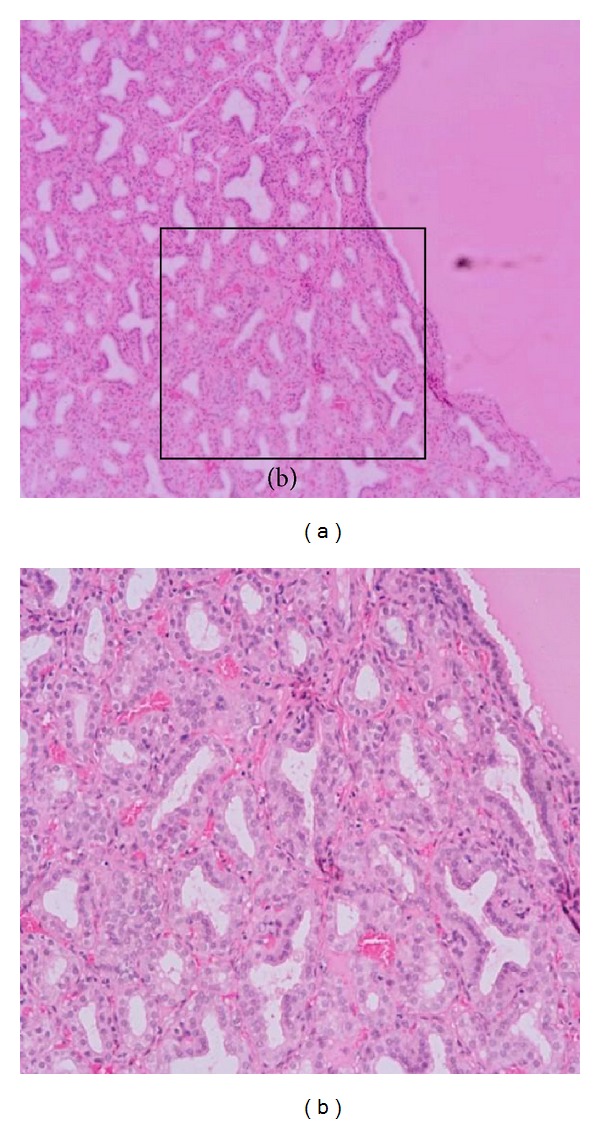
Thyroid sections. (a) A macrofollicular structure contrast with collapsed and folded empty follicular structures. H&E, 80x. (b) Detail of collapsed and folded empty follicular structures in ewes, H&E, 160x.

**Figure 7:**
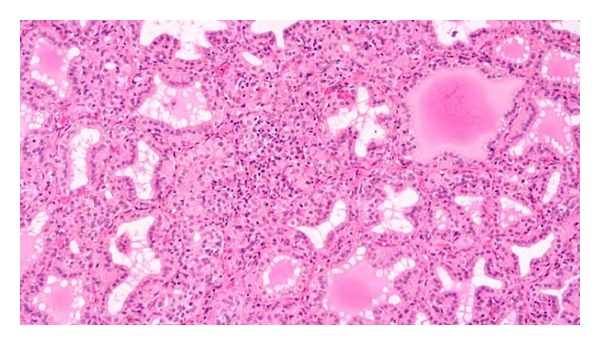
Thyroid sections folded, empty, and vacuolated content follicular structures and a disrupted follicular zone, with pyknotic nucleus in ewes, H&E 160x.

**Figure 8:**
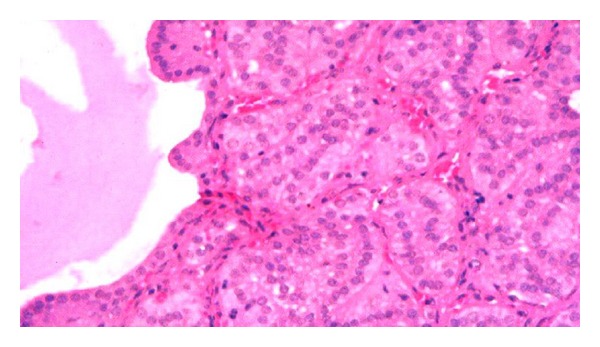
Thyroid sections big follicular structure with a folded wall, collapsed follicles, and pyknotic interstitial nucleus in ewes, H&E, 380x.

**Figure 9:**
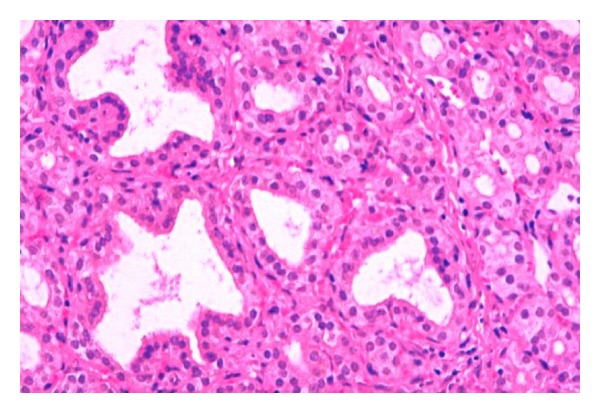
Thyroid sections folded empty follicular structures associated with embryonic aspect rounded follicles. Pyknotic interstitial and follicular nucleus are present in ewes, H&E, 380x.

The diaphragm and myocardium histopathological studies revealed that muscular nucleus proliferation and mononuclear macrophages infiltrate in association with swollen muscular fibers (Figure 5). However, the most conspicuous alterations were observed in the thyroid gland of ewes. There were notorious differences in size, follicular content aspect, staining homogeneous or vacuolated, and empty follicles. Large and dilated follicles, macrofollicles, with flat epithelial cells (goiter aspect) coexisted with much smaller follicles that presented columnar epithelium (embryonic aspect). The most relevant changes included folded follicular epithelial structures, with villous aspect and irregular follicular lumen. Double epithelial layer was observed in some follicles coexisting with collapsed follicular structures observed as cords and nodular columnar epithelial structures. Pyknotic nucleuses were observed in epithelial and fusiform interstitial cells (Figures 6, 7, 8, and 9). Lambs thyroid glands were considered normal; some differences on follicular size were observed only. Histological muscular and thyroid changes were independent of treatment.

## 5. Discussion

Besides agreeing with previous studies showing the important deficit in vegetal and animal selenium levels in most regions of México [19], present results add further support that Se supplementation is critical in these conditions to sustain ruminants' production and thyroid function, similar to other animals and humans [2, 4–8, 13, 19–21]. On the other hand, iodine deficiency and goitrogenic factors have been reported in humans in focalized areas of México only [22, 23]. Animal deficiency has not been reported in México.

According to studies in bovines and ewes [8, 12, 13, 21], present results demonstrate clearly the importance of Se supplementation to maintain Se and T_3_ homeostasis in both pregnant ewes and their offspring. Rock et al. [13] demonstrated this condition in pregnant ewes but T_3_ only presented a tendency to have higher levels in their lambs. Differences between this work and our results may be due to original levels of Se in ewes, the source of Se supplementation, breed and age of experimental ewes, and in the fact that they evaluated 12 h pospartum experimental lambs only. Rock et al. [13] used crossbreed wool sheep (Rambouillet × Polypay); in contrast, in this work, crossbreed hair ewes (Pelibuey × Katahdin) were used; this difference may affect Se and T_3_ thermometabolism requirements.

Our data show that lambs that were born from Se-supplemented ewes and took naturally colostrum and milk from their dams had serum T_3_ significantly higher than those from nonsupplemented ewes. Moreover, serum T_3_, plasma, and milk Se concentration were positively correlated in supplemented and nonsupplemented ewes as well as in lambs that were born from supplemented ewes. In contrast, this correlation was negative in newborns from nonsupplemented ewes. These data strongly suggest that nonsupplemented ewes reduce Se transfer to milk and to the offspring, diverting Se reserves primarily to maintain their own Se and thyroid homeostasis. These results agree with those of other researchers [8, 11] and confirm the hierarchical importance of selenoenzyme synthesis in the maternal organism [5, 24, 25].

High mortality rates in newborns lambs from nonsupplemented ewes than in those born from supplemented ewes have been reported [26]. In this context, hypothermia is one of the major causes of newborn lamb mortality and that T_3_ is essential to the synthesis of uncoupling protein necessary for the thermogenic activity of brown adipose tissue [27, 28]. Nevertheless Rock et al. [13] did not demonstrate effects of Se supplementation on thermometabolism of experimental lambs. This data support the proposal of maintaining ewes Se supplementation throughout the lactation period. Significant increase in T3 in ewes and lambs of Se supplemented group against the control one may be attributed to increase in the activity of Se dependent deiodinase enzymes [29].

The significant positive relationship between maternal plasma Se and serum T_3_ concentrations and the significant decrease in T_3_ levels towards parturition in the control group indicates the importance of maternal Se supplementation to maintain Se and T_3_ homeostasis in pregnant ewes. These results agree with Rock et al. [13] and Rowntree et al. [21] observations in cows as well as with the well-known transplacental Se transference [17, 30] and its beneficial effects throughout colostrum and milk [20, 31].

According to previous studies in ewes and cows [12, 13], lambs born from Se supplemented ewes had serum T_3_ significantly higher than those born from nonsupplemented ewes. At the 8th week postpartum, no significant differences between lambs born from Se supplemented and control groups were observed, probably associated with decrease in milk Se concentration at this moment [31]. Head et al. [32] found that T3 in newborn lambs increased since the 4th day postpartum and then decreased on the 84th day and then began to rise. In this work, T3 level began to decrease on the 5th week postpartum and rose again on the 8th week. These findings suggest that T3 levels were elevated during lambs active growth associated with high milk production of the dams and then decreased and began to rise again when lamb initiated forage intake. This enforces the importance of lambs Se supplementation before the 8th week to maintain plasma Se level and consequently T3 activity.

Calves born from Se supplemented dams had higher levels of T3 than those born from nonsupplemented [12]. These results agree with the ones of the present study. Nevertheless, Rowntree et al. [21] found that maternal Se supplementation only until calving did not influence neonatal calf thyroid hormone concentrations.

No relationships between muscular and thyroid histopathological changes with Se supplementation were observed. Probably lesions occurred in a previous experimental Se deficiency period and persist as a scar; nevertheless, ewes were supplemented. Early reproductive activity and twin gestation in the ewes used for the experiment may cause Se deficiency condition and serious lesions described. Short supplementation period before animals were sacrificed probably was insufficient to lesions resolution. Observed lesions were similar to experimental animals previously described with Se and iodine deficiencies [5, 33] and suggested a combination between degenerative necrotic lesions and hypertrophic compensatory changes. These severe thyroid pathological changes have not been previously described in ewes or other domestic animals. Human studies in México did not consider Se deficiency as a goitrogenic factor [22, 23]. The usage of sheep as a model should be considered as Se status of experimental animals to evaluate thyroid function [34, 35].

## 6. Conclusion

Pre- and pospartum Se supplementation is essential to maintain Se and T_3_ homeostasis during late pregnancy and postpartum period in ewes and their lambs until the 8th week of age.

## Figures and Tables

**Table 1:** Diet supplied to ewes during experimental period (kg/head/day).

Item	Gestation	Lactation
Roughage	0.4	0.5
Alfalfa	1.3	0.7
Soy-bean	0.2	0.4
Ground corn	0.3	0.9
Mineral salt	0.006	0.007
Water	*Ad libitum *	*Ad libitum *
Crude protein	15%	15%
Energy (Mcal/kg)	2.3	2.7
